# Solubilized chlorin e6-layered double hydroxide complex for anticancer photodynamic therapy

**DOI:** 10.1186/s40824-022-00272-8

**Published:** 2022-06-11

**Authors:** Young-um Jo, HyunJune Sim, Chung-Sung Lee, Kyoung Sub Kim, Kun Na

**Affiliations:** 1 Department of Biotechnology, The Catholic University of Korea, 43 Jibong-ro, Wonmi-gu, Gyeonggi-do, Bucheon-si, 14662 Republic of Korea; 2 Department of Biomedical-Chemical Engineering, The Catholic University of Korea, 43 Jibong-ro, Wonmi-gu, Gyeonggi-do, Bucheon-si, 14662 Republic of Korea; 3 Department of Pharmaceutical Engineering and Biotechnology, Sun Moon University, Chungcheongnam-do 31460, Asan-si, Republic of Korea

**Keywords:** Layered double hydroxide, Mono-layered double hydroxide, Anticancer photodynamic therapy, Photosensitizer, Solubilization

## Abstract

**Background:**

Layered double hydroxides (LDHs) are one type of 2-dimensional material with unique structure and strongly positive surface charge. Particularly, LDHs can be exfoliated by mono-layered double hydroxides (MLHs) as a single layer, showing an increased surface area. Therefore, there is a large focus on LDHs for drug delivery applications. Furthermore, most photosensitizers are hydrophobic that they cannot be soluble in aqueous solvents. Herein, we designed a simple way to solubilize hydrophobic photosensitizers by MLH with electrostatic interactions for anticancer photodynamic therapy (PDT), which has tremendous therapeutic advantages. The photosensitizer solubilized via loading on the MLH exhibited fluorescence and singlet oxygen-generation activities in aqueous solvent without chemical modification, resulting in photo-mediated anticancer treatment.

**Methods:**

Negatively charged hydrophobic photosensitizers, chlorin e6 (Ce6) were solubilized by loading on the MLHs through the electrostatic interaction between positively charged MLHs. MLH/Ce6 complexes evaluated for physico-chemical characterization, pH-sensitive release property, in vitro photocytotoxicity, and in vivo tumor ablation.

**Results:**

The photosensitizer solubilized via MLH exhibited fluorescence intensity and singlet-oxygen generation activities in aqueous solvent without chemical modification, resulting photocytotoxicity in cancer cells. The encapsulation efficiency of Ce6 increased to 21.2% through MLH compared to 0.6% when using LDH. In tumor-bearing mice, PDT with solubilized MLH/Ce6 indicated a tumor-suppressing effect approximately 3.4-fold greater than that obtained when Ce6 was injected alone.

**Conclusions:**

This study provided the solubilized Ce6 by the MLH in a simple way without chemical modification. We demonstrated that MLH/Ce6 complexes would have a great potential for anticancer PDT.

**Supplementary Information:**

The online version contains supplementary material available at 10.1186/s40824-022-00272-8.

## Background

Layered double hydroxides (LDH) are a class of positively charged ionic layered compounds. Their structure is expressed as Brucite-like layered (MgOH_2_) wherein a portion of the trivalent ions are incorporated into the preformed divalent metal ion layer isomorphous substitution, resulting in a positively charged layer. The chemical formula for LDH is [M_1-x_^2+^M_x_^3+^(OH)_2_]^x+^[A_x/c_^c−^]^x+^‧zH_2_O (M^2+^ and M^3+^ mean divalent and trivalent metal cations, e.g. Mg^2+^, Ni^2+^, Zn^2+^, Al^3+^, Mn^3+^, and Fe^3+^, x values: 0.2 ≤ x ≤ 0.33, A^c−^ are intercalated inorganic or organic anions, e.g. CO_3_^2−^, NO_3_^−^, and SO_4_^2−^) [1–3]. LDH has been applied in a broad range of materials, such as, fire retardants (water content), oxygen generation (Ni–Fe LDHs), ion exchangers (intercalation of the interlayer space), and polymer/LDH nanocomplexes [4–8]. Moreover, LDHs have been developed as drug carriers in a wide range of medical fields. The coordination bond of hydroxyl groups with metal ions makes LDHs biodegradable in acidic conditions, releasing biocompatible cations and H_2_O due to biodegradation of LDH, minimizing the risk of long-term bioaccumulation and enhancing therapeutic effects [9, 10]. Additionally, LDH is used as a stabilizer of emulsions because a 3 dimensional network is formed between LDHs and oil droplets, resulting in the prevention of oil droplet coalescence [11]. However, LDH has limited access to the interlayer space, causing low interaction activity of LDH with other molecules, e.g. polymers, drugs, and proteins, resulting in low amounts incorporated into the interlayer space [12]. To overcome these limitations, we exfoliated LDHs into a single layer called monolayered double hydroxide (MLH). Exfoliation to provide a large surface area allows for strong interaction of a single layer LDH with other molecules [13, 14]. One of the major drawbacks to the biomedical application of exfoliation of LDH nanosheets is that conventional exfoliation methods are used to create and maintain exfoliation states, using organic solvents (e.g. formamide) that are harmful to biological systems [15].

Photodynamic therapy (PDT) has received much attention for cancer therapy due to its noninvasiveness and efficient therapeutic efficacy. In the process of PDT, a photosensitizer (PS) is administered and the laser irradiates only tumor lesions, resulting in the generation of singlet oxygen (^1^O_2_) for the induction of apoptosis or necrosis of cancer cells [16]. PDT is non-invasive and shows the lowest toxicity to normal tissue compared to other cancer therapies. Additionally, there is no distinct PS resistance or easy access to combination with other therapeutic approaches. However, the therapeutic efficacy of PDT is significantly limited due to the insolubility of PS in aqueous solutions. Insoluble PS induces aggregation, so limits ^1^O_2_ generation and fluorescence quantum yields [17]. There were many studies, such as amino acid polymerized PS, mPEG conjugated PS, and polysaccharide conjugated PS to overcome this limitation [18–24]. Additionally, many marketed pharmaceutical PS products (Photochlor®, Foscan®, and TPCS2a®) use Tween80® or organic solvents [25, 26]. However, Tween80® and organic solvents cause patient hypersensitivity reactions, which are reactions unwanted by the immune system, and peripheral neuropathy, resulting in severe death [27].

**Fig. 1:**
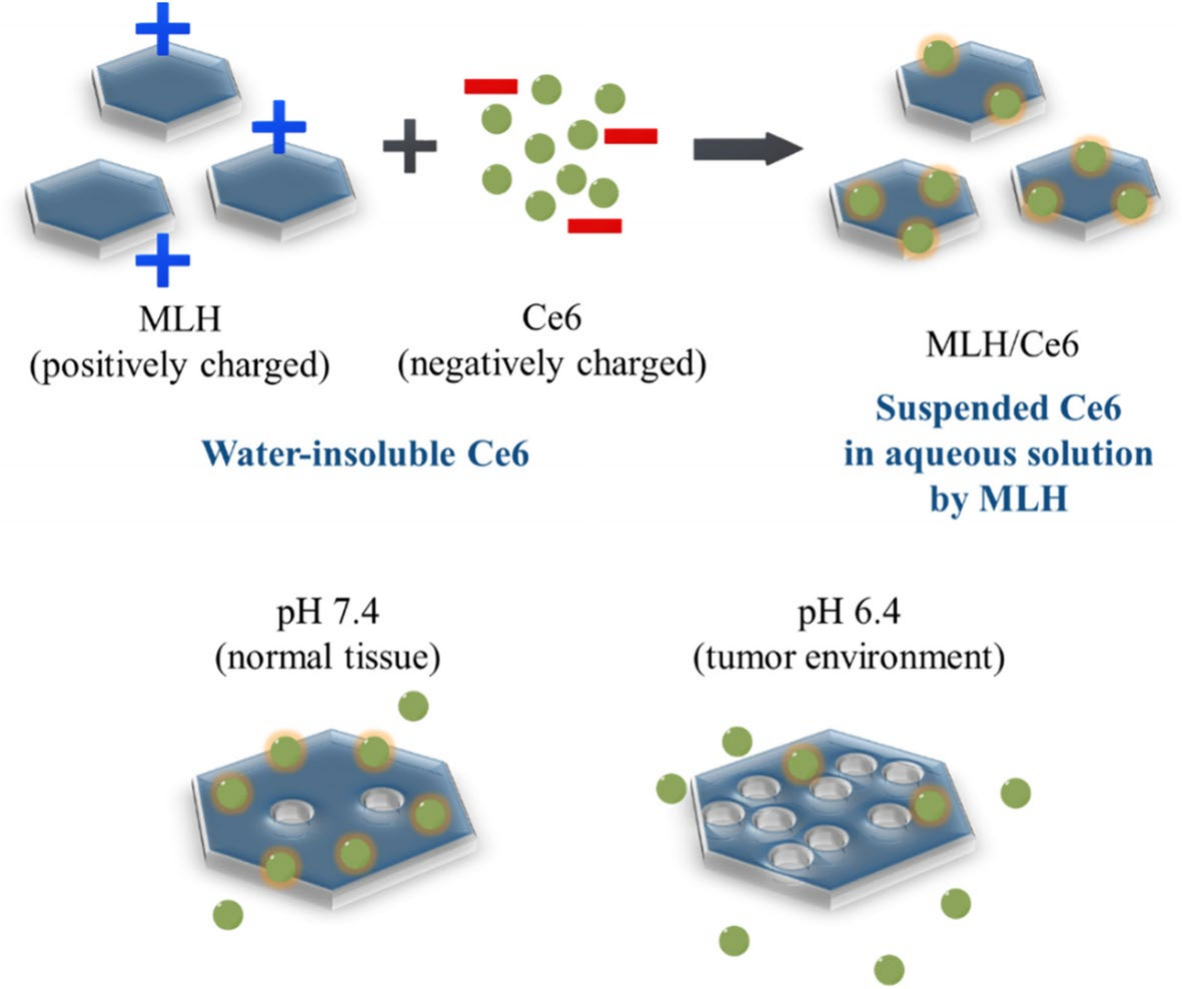
Schematic illustration of MLH/Ce6. The positively charged MLH interacts with Ce6 through electrostatic forces to generate MLH/Ce6

To advance a photodynamic agent for PDT, overcoming the aforementioned drawbacks, we herein developed a facile preparation of solubilized PS (chlorin e6; Ce6) with MLH (MLH/Ce6) using biocompatible lactic acid to exfoliate multilayer LDHs into MLHs (Fig. 1). We hypothesized that the MLH as an emulsion stabilizer could solubilize negatively charged hydrophobic PSs such as Ce6 through the electrostatic interaction between positively charged MLH and Ce6. Physico-chemical characterizations, pH-sensitive release property, in vitro photocytotoxicity, and in vivo tumor ablation of MLH/Ce6 were evaluated and reported. This biocompatible nano-PS based on MLHs can be considerably effective for anticancer PDT.

### Experimental

#### Chemicals and reagents

Aluminum L-lactate, magnesium L-lactate hydrate, sodium hydroxide, DL-lactic acid, anhydrous ethanol, thiazolyl blue tetrazolium bromide (MTT), dimethyl sulfoxide (DMSO), phosphate buffered saline (PBS), Tween 20, and albumin from human serum (A3782; globulin and fatty acid free, lyophilized powder) were purchased from Sigma-Aldrich (St. Louis, Missouri, USA). Chlorin e6 (Ce6) was obtained from Frontier Scientific (Logan, Utah, USA). Singlet oxygen sensor green (SOSG, S36002) was purchased from Thermo Fisher Scientific (Waltham, MA, USA). Float-A-lyzer was purchased from Spectrum Laboratories, Inc. (Rancho Dominguez, CA, USA). A murine colon cancer CT-26 cell line was obtained from the Korean Cell Line Bank (no. 80009, KCLB). DMEM high glucose, Fetal bovine serum (FBS), antibiotics (penicillin/streptomycin), Dulbecco’s phosphate-buffered saline (DPBS), and trypsin–EDTA were purchased from Fisher Scientific (Hampton, NH, USA).

#### Synthesis of MLH

Monolayered double hydroxides (MLHs) were prepared under nitrogen gas as formerly described with slight modifications [28]. DL-lactic acid solution (0.225 M) was adjusted to pH 10 with 2 N NaOH solution. Fifty milliliters of mixed aluminum and magnesium L-lactate solution (0.1 M, Mg:Al = 3:1 molar ratio) were dropped into 50 mL of DL-lactic acid solution. The mixture was stirred and maintained at pH 10 using 2 N NaOH solution. The reaction was performed for 30 min, the centrifugation of the MLH slurry is repeated (3000 rpm, 5 min) and the supernatant is replaced with water using a sealed containers to avoid CO_2_ contamination. Deionized water (D.I water) was used immediately after filtration. The aggregated pellet was resuspended in 100 mL of D.I water and stirred until 2 days. The turbid MLH solution became transparent. The clear MLH solution was lyophilized and stored at -20 °C.

#### Preparation of MLH/Ce6

MLH/Ce6 was prepared by a thin-film hydration method. First, 10 mg of MLH powder was dissolved in 10 mL of anhydrous ethanol. Ten milliliters of MLH solution (1 mg/mL) in ethanol was mixed with 1 mL of Ce6 solution (1 mg/mL) in ethanol and then removed ethanol using a rotary evaporator. The thin film produced in the round-bottom flask was hydrated with 10 mL of D.I water and then sonicated. To remove free Ce6, the sonicated solution was centrifugated at 3000 rpm for 10 min. For further in vitro and in vivo experiments, HSA was selected as a stabilizer, and 10 mL of MLH/Ce6 solution was added to 100 mg of HSA powder. The MLH/Ce6/HSA was diluted 10% (volume/volume) with 10X PBS [29].

#### Characterization

Powder X-ray diffraction of lyophilized MLH was analyzed using MiniFlex600 (Rigaku, Japan) with Cu Kα radiation (λ = 1.5418 Å), operated 40 kV, 30 mA and a scanning rate of 0.1°/min. Transmission electron microscopy (TEM) images were obtained from TEM II with a JEM-2100 ccd camera (JEOLP Ltd, Japan). Optical and fluorescence images were captured by fluorescence-labeled organism bioimaging (FOBI, NeoScience, Suwon, Korea). The fluorescence intensity was confirmed by analysis with NEOimage software (NeoScience, Suwon, Korea).

The amount of Ce6 incorporated into MLH was quantified. Briefly, MLH/Ce6 solution was diluted to 1/10 with DMSO. The Ce6 standard started from 1 to 0.000313 mg/mL in 90% DMSO. The fluorescence of the samples and standard was analyzed with multiplate reader (Bio-Tek, VT, USA) with ex/em 419/670 nm. The Ce6 loading efficiency and loading capacity were calculated with the following equation:

1 $$\mathrm{Loading efficiency }(\mathrm{\%}) = \frac{Feeding amount of Ce6-Nonencasulated Ce6}{Feeding amount of Ce6}\times 100$$

2 $$\mathrm{Loading contents }(\mathrm{\%}) = \frac{Total weight of encapsulated Ce6}{Total MLH/Ce6 weight}\times 100$$

The size and ζ-potential of MLH/Ce6 were measured using a Zetasizer Nano ZS (Malvern Instruments, UK). Zetasizer was performed by automatic sampling times and analysis at room temperature.

#### Singlet oxygen generation test

The singlet oxygen generation test was performed with singlet oxygen sensor green (SOSG, S-36002) as a probe. Free Ce6 was dissolved in D.I water to 8 μg/mL and MLH/Ce6 solution was diluted to 8 μg/mL of Ce6 concentration. The SOSG solution (2 mM) was added to the Ce6 solution and diluted MLH/Ce6 solution. The resulting mixtures were exposed to 670 nm laser radiation (20 mW/cm^2^, fiber-coupled laser system, LaserLab, Korea). Fluorescence of SOSG were measured with a spectrofluorometer (RF-5301, Shimadzu, Japan) at ex/em 494/534 nm.

#### Chlorin e6 release profile

The Ce6 release profiles of MLH/Ce6 were obtained with a dialysis membrane. Briefly, 1 mL of MLH/Ce6/HSA solution was transferred to the inner space of Float-A-lyzer G2 (molecular weight-cutoff 100 K) and 5 mL of pH 6.4 and 7.4 PBST (0.01 M, 0.1% w/v of TWEEN® 20) was transffered into the outer space of the dialysis tube. The resulting dialysis bags were stored in a water shaker at 50 rpm and 37 °C. At time points (0, 1, 3, 6, 12, 24, 48, and 72 h), outer buffer was taken into a conical tube and filled with PBST. The fluorescence of released Ce6 was detected with a multiplate reader.

#### Cell culture

A murine colon cancer CT-26 cell line was obtained from the Korean Cell Line Bank (no. 80009 KCLB). CT-26 cells were cultured in DMEM high containing 10% FBS, antibiotics (100 IU/mL penicillin/ 100 μg/mL streptomycin). Cells were incubated at 37 °C with humidified 5% CO_2_ and subcultured in new media every 2–3 days with DPBS and trypsin–EDTA.

#### Cellular uptake

CT-26 cells were seeded onto 6-well plates at a density of 1 × 10^7^ cells per well. After overnight incubation, the cell cultures were incubated with a medium containing MLH/Ce6. The cells were incubated for the following time points as 1, 2, and 4 h at 37 °C at a Ce6 concentration of 1 μg/mL and washed twice with DPBS, followed by cell detachment for flow cytometry (Becton Dickinson Biosciences, USA). Each sample was counted by 1 × 10^4^ cells (gated events). The fluorescence of Ce6 was detected using logarithmic settings with an emission wavelength of 620 nm. Each experiment was confirmed by analysis with FlowJo software (FlowJo LLC, United States).

#### In vitro cytotoxicity evaluation

Cells were seeded onto 48-well plates at a density of 1 × 10^6^ cells per well. Following overnight incubation, the cell cultures were incubated with a medium containing MLH/Ce6. The cell cultures were incubated for 4 h at 37 °C at various Ce6 concentrations and washed two times with DPBS. The Serum-containing medium was added to each well, followed by exposure to 670 nm laser radiation (50 mW/cm^2^, 40 s). After treatment with MTT solution (0.2 mg/mL) and incubation for 4 h, cell viability was recorded by a multiplate reader at a wavelength of 570 nm. To optically estimate cell viability, we used the Live & Dead assay kit (Molecular Probes, U.S.A). The dyes in this kit were 2 μM Calcein acetoxymethyl ester (calcein-AM, green fluorescent dye) and 4 μM ethidium homodimer (EthD-1, red fluorescent dye). CT-26 cells were seeded onto 6-well cell culture plates at a density of 5 × 10^5^ cells per well. CT-26 cell culture plates were incubated overnight at 37 °C in 5% CO_2_. Following 12 h, the cultured medium was removed, and the cells were washed twice with DPBS. Laser irradiation (50 mW/cm^2^, 40 s) was performed using a 670 nm laser source. After laser irradiation, the cells were incubated in a complement medium for 48 h. To confirm cell viability, the cells were observed through confocal laser scanning microscopy (CLSM, LSM 510 Meta; Zeiss, Germany) [30]. To detect cellular ROS, we performed DCFDA assay. CT-26 cells were seeded in 96-well cell culture plates with 1 × 104 cells per well. The cells were incubated overnight at 37 °C in 5% CO2. After we removed the cultured medium and washed with DPBS. The cells were incubated with a medium containing MLH/Ce6 (pH 7.4, 6.4; concentration of Ce6: 1 μg/mL). Following 4 h, the medium was washed twice with DPBS and replaced 20 μM DCFDA solution. Thirty minutes later, the solution was replaced with DPBS and laser irradiated (50 mW/cm2, 40 s). the fluorescence of DCFDA was measured by a multiplate reader.

#### In vivo animal experiments

All animal experiments were approved by the Institutional Animal Care and Use Committee (IACUC) of the Catholic University of Korea in accordance with the “Principles of Laboratory Animal Care,” NIH publication no. 85–23, revised in 1985. Tumor accumulation and antitumor activity of MLH/Ce6 were evaluated using 6-week-old BALB/c mice (Orient Bio, Seongnam, South Korea).

#### In vivo tumor accumulation evaluation

In vivo Tumor accumulation of MLH/Ce6 was evaluated with CT-26 bearing tumor mouse model. Briefly, six-week-old male BALB/c mice were transplanted subcutaneously with CT-26 (1 × 10^5^ cells per mouse). Tumor accumulation was conducted when the transplanted tumor reached approximately 10 mm^3^ in volume. The mouse model was administered a 0.2 mg/kg dose of MLH/Ce6 via the tail vein (*n* = 3). The mice were imaged by FOBI animal fluorescent optical imaging at various time points (0, 1, 3, 6, 12, and 24 h). After all experiments ended, the animals were euthanized with CO_2_ gas.

#### In vivo antitumor photodynamic therapy

To study the tumor inhibition effect of MLH/Ce6, CT-26 tumor bearing mice were randomized into five groups of 5 animals per group. When the tumor volumes reached approximately 100 mm^3^, the groups of mice were intravenously injected with 100 μL of saline(control group), free Ce6(-), free Ce6( +), MLH/Ce6(-), and MLH/Ce6( +) (2 mg/kg of Ce6). At 12 h of administration, the free Ce6( +) and MLH/Ce6( +) groups were exposed to a 671 nm laser (100 J/cm^2^). Each group was treated at day zero and on the first day. The volume of the tumor and body weight were recorded for 14 days after the first injection.

## Result

### Synthesis and characterization of mono-layered double hydroxide

**Fig. 2:**
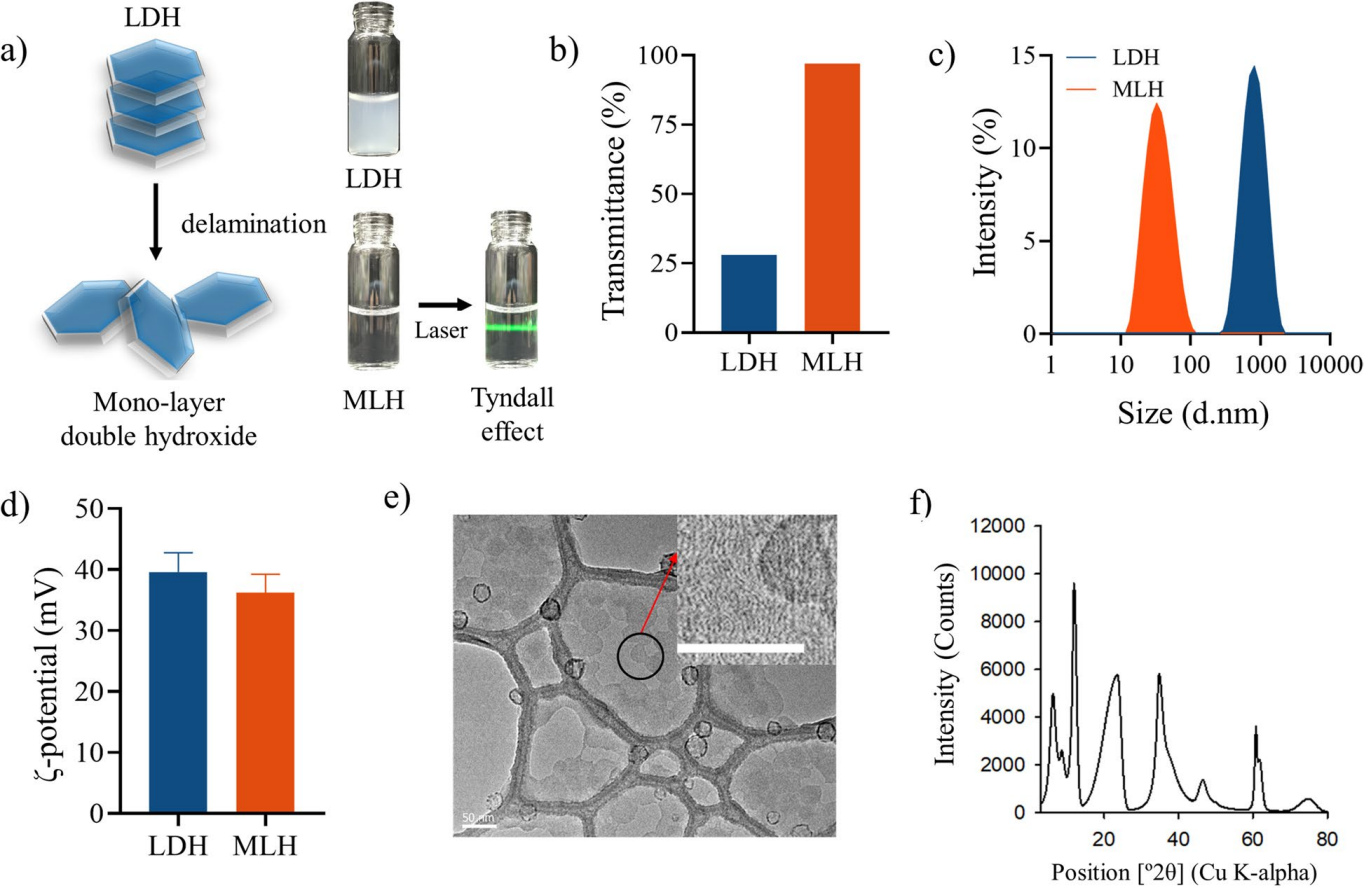
Delamination of LDH into the MLH and characterization of LDH and MLH. **a** Schematic illustration of LDH and MLH. Photographs of the Tyndall effect in aqueous solutions of LDH and MLH (concentration of LDH and MLH = 1 mg/mL). **b** Transmittance of LDH and MLH in aqueous solutions. **c** Size distribution and (**d**) ζ potential of LDH and MLH by DLS, (**e**) TEM image of MLH (scale bar = 50 nm), (**f**) X-ray powder diffraction patterns of MLH

LDH was fabricated by the coprecipitation method from a homogeneous solution. MLH was synthesized by exfoliating LDH composed of multiple layers. LDH is opaque whereas MLH became transparent (Fig. 2a). The presence of MLH was demonstrated through the Tyndall effect by light scattering under a laser, which is a simple method to indicate a well-dispersed colloidal solution. Figure 2b shows the formation of MLH using transmittance. The transmittance of LDH was approximately 26%, and the transmittance of MLH was approximately 100%. The average hydrodynamic diameter of MLH was decreased as LDH became exfoliated (Fig. 2c). The average sizes of LDH and MLH were 824 ± 40 nm and 42 ± 2 nm in aqueous solution, respectively. Zeta-potential of LDH and MLH has a cationic surface charge (Fig. 2d). MLH on TEM was hexagonal in shape, and the size was approximately 30 ~ 40 nm (Fig. 2e). The XRD patterns of the MLH indicates sharp distinct peaks, at (003), (006), (009), (015), (018), (110), and (113), that are coincident with intrinsic patterns of LDH in the literature (Fig. 2f) [31].

### Preparation and characterization of monolayered double hydroxide/chlorin e6

**Fig. 3:**
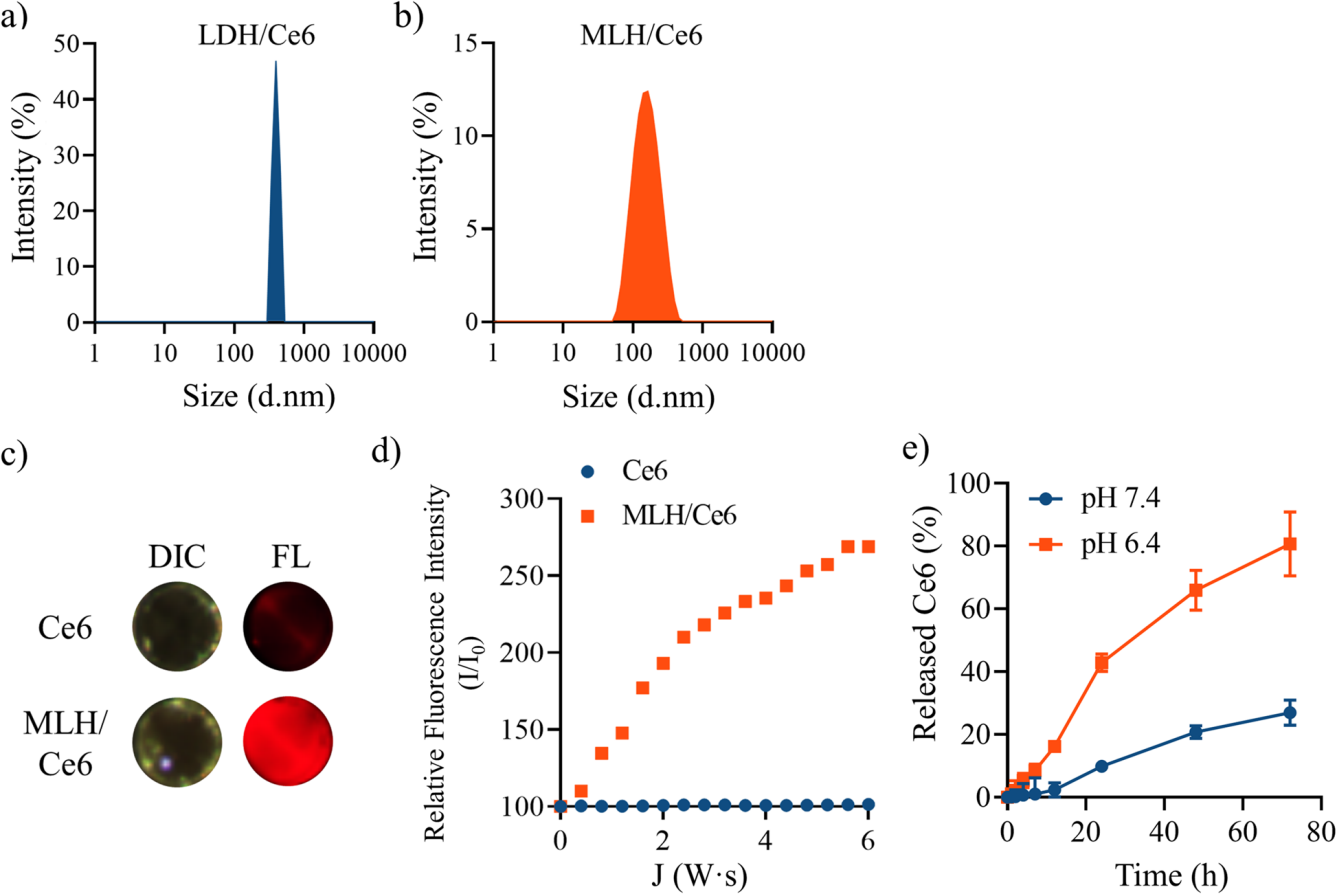
Characterization of LDH or MLH/Ce6. Size distribution of (**a**) LDH/Ce6 and (**b**) MLH/Ce6 in water by DLS, (**c**) Optical and fluorescence images of Ce6 and MLH/Ce6. **d** Singlet oxygen generation profile of Ce6 and MLH/Ce6 by singlet oxygen sensor green, (concentration of Ce6 = 3.5 µg/mL, SOSG = 2 mM, laser power = 20 mW) (**e**) Accumulative Ce6 release profiles of MLH/Ce6 in pH 6.4 and pH 7.4 buffer

LDH/Ce6 and MLH/Ce6 were prepared by thin film hydration. LDH and MLH solutions in anhydrous ethanol were mixed with Ce6 solutions in anhydrous ethanol and the resulting mixture was evaporated, followed by hydration with DI water. The LDH/Ce6 and MLH/Ce6 showed 344.4 ± 58.2 nm and 182.7 ± 23.4 nm of hydrodynamic diameters, respectively (Fig. 3a,b). The LDH/Ce6 and MLH/Ce6 exhibited a positive charge and maintained the size and PDI for at least 5 days (Fig. S1). There was a significant difference in Ce6 loading efficiency and loading contents between LDH/Ce6 and MLH/Ce6. LDH/Ce6 could load Ce6 at only 0.06% of the weight and 2.10% in MLH/Ce6. Furthermore, the loading efficiencies of LDH/Ce6 and MLH/Ce6 were 0.57% and 21.21%, respectively. To confirm Ce6 fluorescence, we visualized Ce6 fluorescence images, although free Ce6 in DI water did not show fluorescence intensity, and MLH/Ce6 displayed a red image, indicating emission of Ce6 fluorescence (Fig. 3c). In PDT, Ce6 is administered as a PS and shows therapeutic efficacy by generating singlet oxygen under laser irradiation of lesions. In this regard, we performed a singlet oxygen generation test with SOSG. The SOSG fluorescence intensity dramatically increased in the dependence of laser power in MLH/Ce6 compared with Ce6 (Fig. 3d). The Ce6 release contents from MLH/Ce6 were increased at pH 6.4 compared to pH 7.4 (Fig. 3e).

Next, we examined the Ce6 release pattern at pH 6.4 and 7.4. MLH/Ce6 showed different release profiles between the pH conditions. The buffer condition (pH 7.4 and pH 6.4) was selected according to the tumor microenvironment (pH_e_ = 6.4) and physiological system (pH_e_ = 7.4) [32]. The Ce6 release amount of MLH/Ce6 at pH 7.4 at 72 h was approximately 22% whereas the Ce6 release amount of MLH/Ce6 at pH 6.4 at 72 h was 83% (Fig. 3e).

### In vitro cellular uptake and anticancer efficacy

**Fig. 4:**
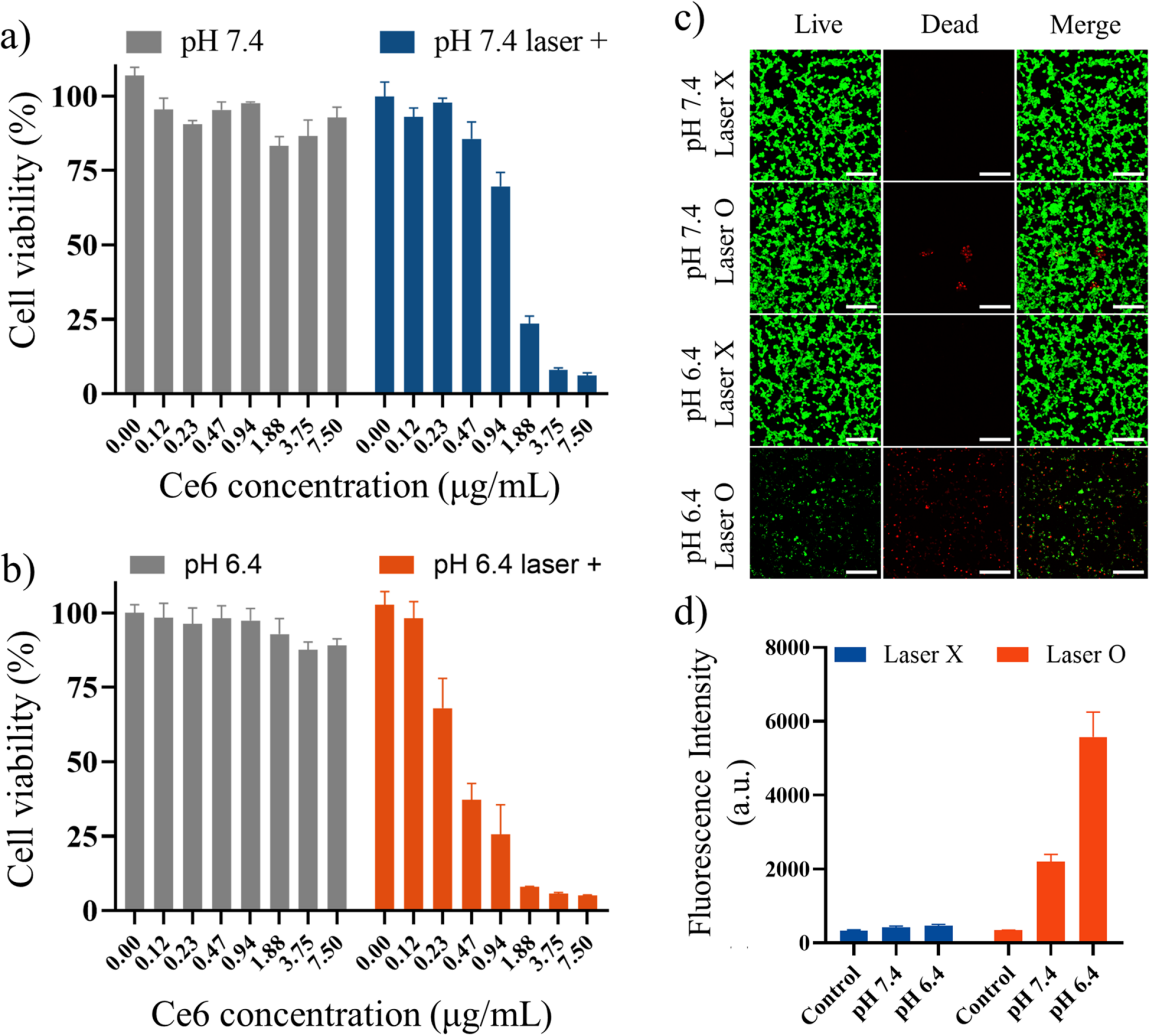
Cell viability of MLH/Ce6 depending on pH conditions. CT-26 cell viability in (**a**) pH 7.4 or (**b**) pH 6.4 in the absence or presence of laser irradiation (laser power = 2 J, incubation time = 4 h), (**c**) CLSM image of the Live&Dead assay. The live cells were stained green. The dead cells were stained red. Scale bar is 100 μm. **d** DCFDA assay of MLH/Ce6 (laser power = 2 J, concentration of Ce6 = 1 µg/mL)

Cellular uptake was evaluated with CT-26 cells depending on the MLH/Ce6 incubation time by detecting Ce6 fluorescence intensity on FACS. As the incubation time increased, the intracellular Ce6 concentration increased for 4 h incubation after MLH/Ce6 treatment (Fig. S2). As a result, we selected an incubation time of 4 h as the highest uptake time. To evaluate the feasibility of using MLH/Ce6 for PDT, an in vitro cell cytotoxicity test was conducted using an MTT assay at pH 7.4 and pH 6.4 in the presence or absence of laser irradiation (Fig. 4a,b). Almost group not shown any cytotoxicity at 0 ~ 4 µg/mL of Ce6 concentration without laser irradiation at pH 7.4 and pH 6.4. However, the group treated under irradiation with MLH/Ce6 concentrations from 1.3 µg/mL displayed approximately 50% viability at pH 7.4. Furthermore, a Ce6 concentration of 3.75 µg/mL in the presence of the laser, which displays no effect on cell viability without irradiation, exhibited therapeutic efficacy against cancer cells of almost 100%. Furthermore, at pH 6.4, cell phototoxicity was stronger than at pH 7.4. In particular, the sample treated group with 0.3 µg/mL of Ce6 and the group at pH 6.4 with laser irradiation showed the strongest therapeutic efficacy, almost 50% cell viability (approximately 85% at pH 7.4 with laser). Based on the MTT assay, a similar trend was observed in the Live&Dead assay {dead cells stained with EthD-1 (red) and live cells stained with calcein-AM (green)}. The CLSM image shows that the pH 6.4 with laser irradiation group has a higher cancer cell death rate than the other groups (Fig. 4c). To determine the cellular ROS generation by MLH/Ce6, DCFDA assay was performed (Fig. 4d). In the MLH/Ce6 treated group (pH 6.4 with laser irradiation), DCFDA fluorescence intensity was higher than that of other groups. Additionally, the cytotoxicity of MLH was evaluated. MLH were treated from 500 to 0.5 µg/mL through a one-half dilution. Although MLH has a powerful positive charge, MLH did not show any toxicity to CT-26 cells (Fig. S3).

### In vivo tumor accumulation

**Fig. 5:**
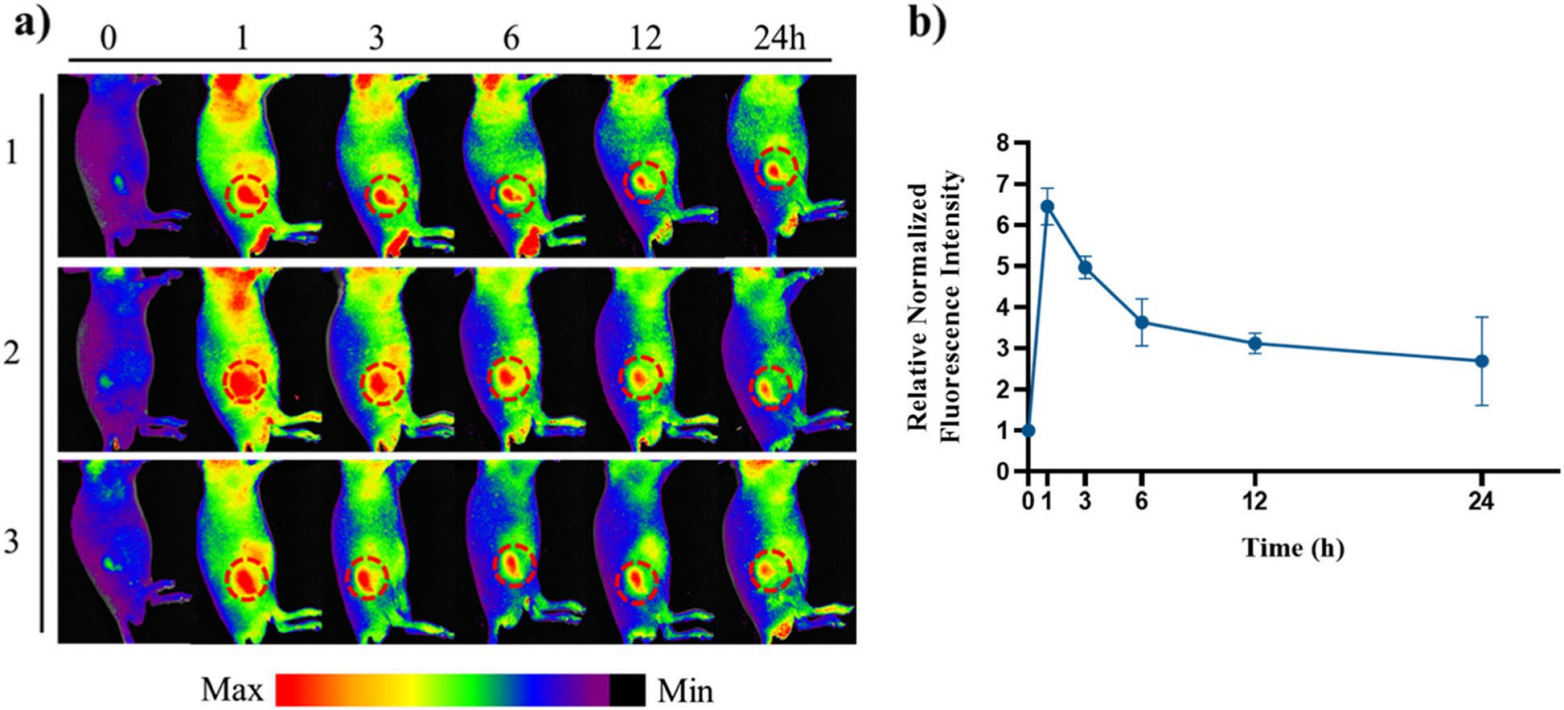
**a** In vivo FOBI image of MLH/Ce6 (3 mg/kg of Ce6) treated CT-26 bearing BALB/c mice. **b** Relative normalized fluorescence intensity of tumor region after MLH/Ce6 injection

To investigate the feasibility of MLH/Ce6 for in vivo applications, we administered MLH/Ce6 via the tail vein into CT-26 tumor-bearing BALB/c nude mice and monitored the accumulation of MLH/Ce6 with FOBI animal fluorescent optical imaging (Fig. 5a). The fluorescence intensity of Ce6 appeared at the tumor site in mice at 1 h postinjection. Additionally, it was retained until 24 h after intravenous injection (Fig. 5b).

### Anticancer PDT

**Fig. 6:**
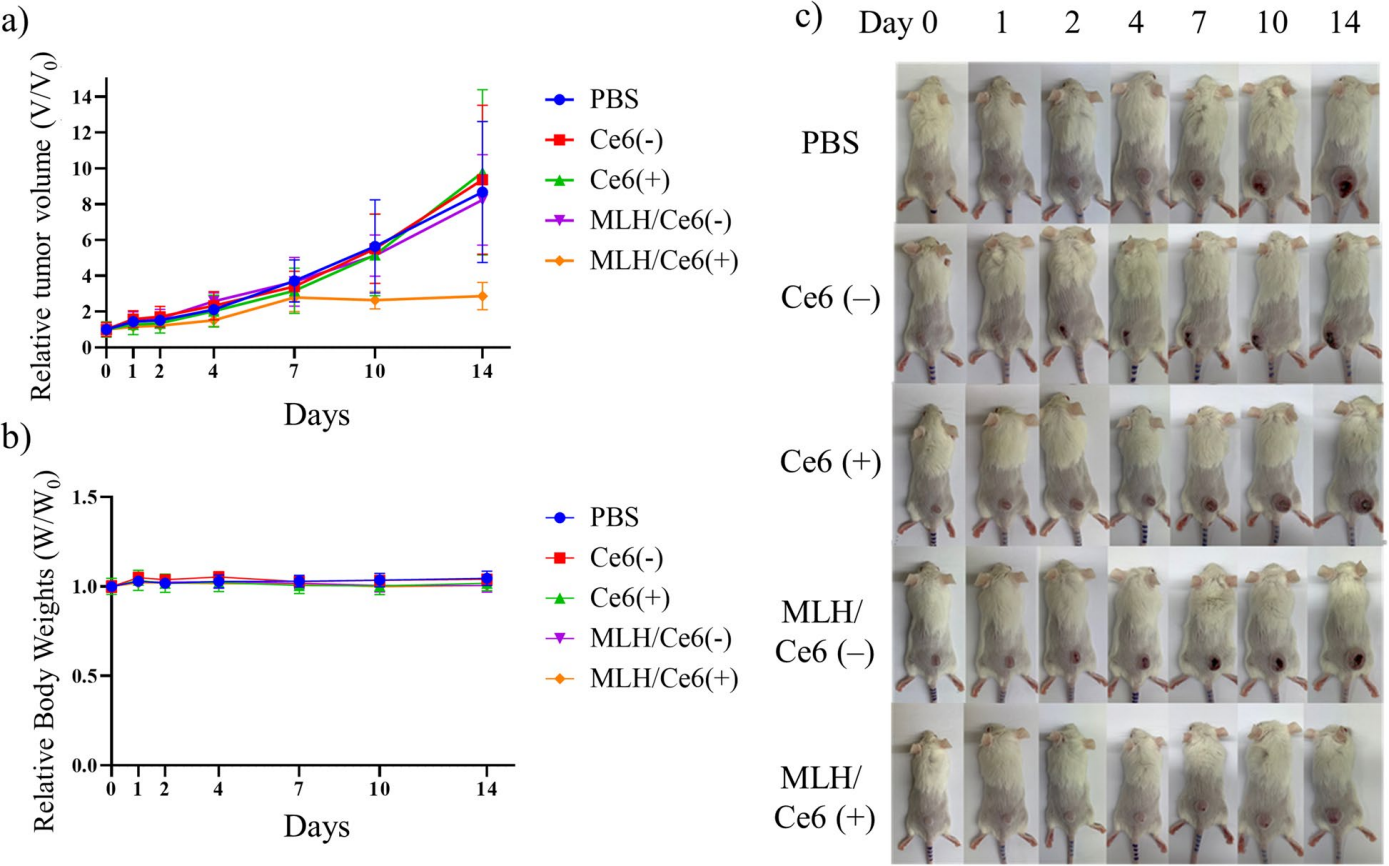
In vivo PDT effect of MLH/Ce6. **a** tumor growth curves, and (**b**) body weight curves of mice with the various treatments. Each value was normalized to their initial sizes (*n* = 5 per group). **c** Corresponding images of tumor-bearing mice taken on day 14 after treatment

Based on tumor accumulation, the in vivo antitumor efficiency of MLH/Ce6 was evaluated in CT-26 tumor-bearing Balb/c mice. When the inoculated tumor size reached 100 mm^3^, saline, free Ce6, and MLH/Ce6 (2 mg/kg of Ce6) was intravenously injected via the tail vein. After 12 h postinjection periods, the tumor tissues were irradiated with a red laser (670 nm, 100 J/cm^2^) and the tumor size was measured for 14 days. In Fig. 6a,c, only tumor tissue treated MLH/Ce6 and irradiated with the laser showed antitumor inhibition effects due to, the excellent phototoxicity. However, free Ce6 treated mice did not show conspicuous phototoxicity in tumor tissue. In contrast, MLH/Ce6 delivered well-solubilized Ce6 to tumor tissues while preventing Ce6 aggregation, which induced excellent therapeutic effects. Furthermore, there were no noticeable changes in body weight in any group during the investigation (Fig. 6b).

## Discussion

In this work, MLH was synthesized by exfoliating multilayered LDH which prepared by the coprecipitation method from a homogeneous solution. The high transmittance and tyndall effect of MLH solution demonstrated that MLH successfully exfoliated from LDH. The XRD patterns of the MLH appeared coincident with intrinsic patterns of LDH in the literature. Based on the overall data, LDH was confirmed to be successfully exfoliated and a two-dimensional material with a positive charge suitable for interacting with negatively charged Ce6 was prepared. The formation of MLH/Ce6 occurs by the electrostatic interaction between positively charged MLH and negatively charged Ce6 with carboxylic groups. LDH/Ce6 and MLH/Ce6 were prepared by thin film hydration. LDH and MLH solutions in anhydrous ethanol were mixed with Ce6 solutions in anhydrous ethanol and the resulting mixture was evaporated, followed by hydration with DI water. The hydrodynamic diameters of LDH/Ce6 and MLH/Ce6 showed both were prepared well. The higher loading efficiency and contents of MLH/Ce6 than those of LDH/Ce6, because Ce6 could not access the inner space of LDH. In this result, MLH was exfoliated from LDH so there was a greater chance for Ce6 to interact with MLH due to their increased surface area. Therefore, we performed further studies except for LDH/Ce6.

The fluorescence intensity of Ce6 in the MLH/Ce6 indicates facile solubilization of Ce6 with only a thin film without any chemical modification due to Ce6 being significantly hydrophobic so that it is quenched in an aqueous solution, resulting in no fluorescence intensity in DI water. We estimated singlet oxygen generation ability of MLH/Ce6 for PDT. The MLH/Ce6 resulted in a much higher SOSG fluorescence intensity than free Ce6. This result suggested singlet oxygen generation ability of MLH/Ce6 was enhanced to solubilize Ce6 by a facile method developed in this study. In Ce6 release test, buffer condition (pH 7.4 and pH 6.4) was selected according to the tumor microenvironment (pHe = 6.4) and physiological system (pHe = 7.4). The Ce6 release rate increased in pH 6.4. This result takes advantage of the prevention of Ce6 leakage. The administered MLH/Ce6 traveled in the blood to reach the tumor. For this journey, MLH/Ce6 can slowly release Ce6 in blood circulation (pH 7.4) until the tumor arrives, thus leading to minimal Ce6 loss. Therefore, MLH/Ce6 can maximize the delivery of Ce6 to tumor lesions.

Through cellular uptake test, we selected an incubation time of 4 h as the highest uptake time for further in vitro studies. The cell viability of MLH/Ce6 with or without laser irradiation was conducted at pH 7.4 and 6.4 conditions for anticancer PDT. In both pH 7.4 and 6.4 showed no effect on cell viability without irradiation. However, at pH 6.4, cell phototoxicity was stronger than at pH 7.4 which exhibited therapeutic efficacy against cancer cells at pH of tumor microenvironments of almost 100%. At Live&Dead assay, MLH/Ce6 with laser irradiation at pH 6.4 had significant different with other groups which means the group at pH 6.4 with laser irradiation showed the strongest therapeutic efficacy. The cell viabilities of MLH were almost same with non-treated groups. Despite highly positive charge of MLH, MLH did not show any toxicity to CT-26 cells. This low toxicity indicated that MLH did not influence the phototoxicity of MLH/Ce6. The low toxicity of MLH is especially meaningful, even though almost positively charged carriers for genes and proteins are well known as highly cytotoxic materials. Consequently, MLHs have great potential as drug delivery carriers for anionic materials (e.g. genes and proteins) with low cytotoxicity, including therapeutic efficacy as anticancer agents.

In vivo MLH/Ce6 tumor accumulation test with CT-26 inoculated mice showed fluorescence intensity at tumor lesion maintained 1 h to 24 h. These results demonstrated that MLH/Ce6 can effectively reach the tumor site after systemic circulation because of the ability of MLH to capture Ce6 based on release patterns. This is the different result of that small molecule PS is rapidly degraded or eliminated from the tumor microenvironment by reticuloendothelial cells [33]. According to previous studies, nanosized materials can reach the tumor site by enhanced permeability and retention (EPR) effects, which indicates that molecules of certain sizes tend to accumulate in the tumor site much more than in normal tissue [34–36]. These properties of nanoparticles not only improve the solubility of PS but also selectivity to cancer tissue. Furthermore, this result indicated the possibility of imaging tumor lesions. Thus, MLH/Ce6 has the potential to track and visualize tumors as well as the feasibility of anticancer therapy.

To evaluate the in vivo antitumor efficiency of MLH/Ce6, we administered MLH/Ce6 into the CT-26 tumor-bearing mice. There were the smallest tumor volumes in MLH/Ce6 with laser irradiation groups. These results indicated that MLH/Ce6 delivered well-solubilized Ce6 to tumor tissues while preventing Ce6 aggregation, which induced excellent therapeutic effects. The body weight of mice showed no significant differences in all groups, so MLH/Ce6 did not cause any side effect, and the anticancer effect of MLH/Ce6 is due to phototoxicity, not caused by the toxicity of the carrier.

## Conclusions

In this paper, we used MLH for solubilization of Ce6 (MLH/Ce6). MLH provided a simple way to solubilize Ce6 with higher Ce6 loading efficiency and loading contents than LDH. MLH/Ce6 displayed a narrow size distribution and Ce6 fluorescence intensity under aqueous conditions. MLH/Ce6 generated singlet oxygen resulting in the phototoxicity, which kills tumor cells under laser irradiation at a concentration that did not influence cell viability without laser irradiation. Additionally, MLH/Ce6 showed the accelerated release of Ce6 at tumor extracellular pH levels (pH_e_ = 6.4) compared with normal tissue conditions (pH_e_ = 7.4), resulting in minimized side effects in blood circulation and enhanced therapeutic efficacy in the tumor region. Lastly, MLH/Ce6 accumulated in the tumor lesion in the CT-26 bearing tumor animal model. Due to their effective generation of ^1^O_2_ and accumulation in tumor tissue, MLH/Ce6 exhibited excellent anticancer ability in the mouse model. Therefore, MLH can be considered a potential solubilizer for negatively charged hydrophobic drugs and a low toxicity carrier for various drugs such as negatively charged molecules, DNA, and RNA.

## Supplementary Information

**Additional file 1: Fig. S1.** (a) ζ-potential of dispersed Ce6, LDH/Ce6, and MLH/Ce6. (b) hydrodynamic size and PDI of MLH/Ce6 with incubation at R.T. (n=3, 1~5 days). **Fig. S2.** Cellular uptake of MLH/Ce6 against CT-26 cells depending on the incubation time using flow cytometry (concentration of Ce6 = 1 µg/mL). **Fig. S3.** Cell viability test of MLHs against CT-26 cells at various concentration (incubation time = 24 h).

## Data Availability

Data sharing is not applicable to this article.

## Abbreviations

LDH: Layered double hydroxide
MLH: Mono-layered double hydroxide
PDT: Photodynamic therapy
Ce6: Chlorin e6
PS: Photosensitizer
MTT: Thiazolyl blue tetrazolium bromide
DMSO: Dimethyl sulfoxide
PBS: Phosphate buffered saline
SOSG: Singlet oxygen sensor green
FBS: Fetal bovine serum
DPBS: Dulbecco’s phosphate buffered saline
TEM: Transmission electron microscopy
FOBI: Fluorescence-labeled organism bioimaging
FACS: Fluorescence activated single cell sorting
D.I water: Deionized water
HSA: Human serum albumin
XRD: X-ray diffraction
EDTA: Ethylenediaminetetraacetic acid
CLSM: Confocal laser scanning microscopy
EthD-1: Ethidium homodimer
calcein-AM: Calcein acetoxymethyl ester
