# Supplementary material for: Solubilized chlorin e6-layered double hydroxide complex for anticancer photodynamic therapy
Source: Biomater Res. 2022 Jun 11;26:23. doi: 10.1186/s40824-022-00272-8 (PMC9188148; doi:10.1186/s40824-022-00272-8)
Supplement: Supplementary file 1 — Additional file 1: Fig. S1. (a) ζ-potential of dispersed Ce6, LDH/Ce6, and MLH/Ce6. (b) hydrodynamic size and PDI of MLH/Ce6 with incubation at R.T. (n=3, 1~5 days). Fig. S2. Cellular uptake of MLH/Ce6 against CT-26 cells depending on the incubation time using flow cytometry (concentration of Ce6 = 1 µg/mL). Fig. S3. Cell viability test of MLHs against CT-26 cells at various concentration (incubation time = 24 h). [file 40824_2022_272_MOESM1_ESM.docx]

Supporting information

Solubilized chlorin e6-layered double hydroxide complex

for anticancer photodynamic therapy

Young-um Jo^†,1^, HyunJune Sim^†1,2^, Chung-Sung Lee^3^_,_ Kyoung Sub Kim^1^, and Kun Na*^,1,2^

^1^Department of Biotechnology, The Catholic University of Korea, 43 Jibong-ro, Wonmi-gu, Bucheon-si, Gyeonggi do, 14662, Republic of Korea
^2^Department of Biomedical-Chemical Engineering, The Catholic University of Korea, 43 Jibong-ro, Wonmi-gu, Bucheon-si, Gyeonggi do, 14662, Republic of Korea
^3^Department of Pharmaceutical Engineering and Biotechnology, Sun Moon University, Asan-si, Chungcheongnam-do 31460, Republic of Korea

* Corresponding author: Kun Na, Ph.D.

^†^These authors contributed equally to this work.

Tel.: +82-2-2164-4832

Fax.: +82-2-2164-4865

E-mail: [kna6997@catholic.ac.kr](mailto:kna6997@catholic.ac.kr)

*
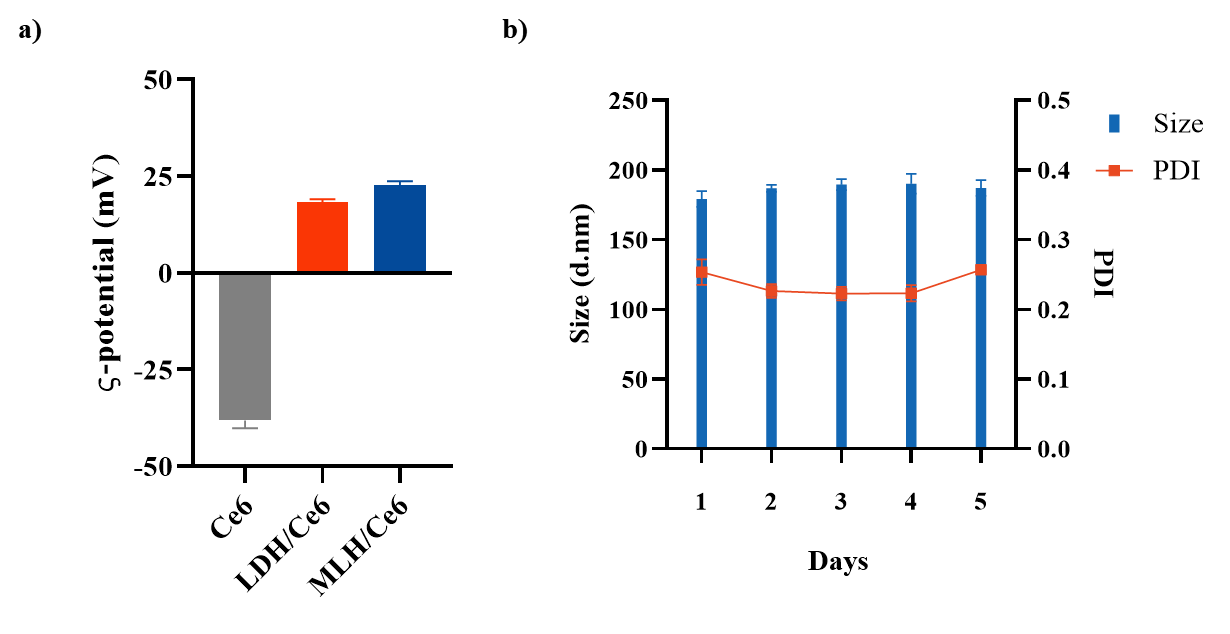
*

**Fig. S1.** (a) ζ-potential of dispersed Ce6, LDH/Ce6, and MLH/Ce6. (b) hydrodynamic size and PDI of MLH/Ce6 with incubation at R.T. (n=3, 1~5 days)


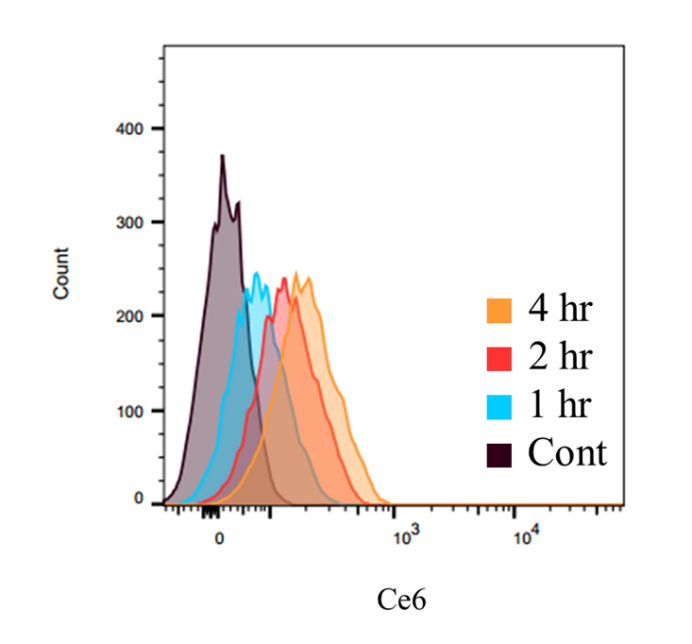


**Fig. S2.** Cellular uptake of MLH/Ce6 against CT-26 cells depending on the incubation time using flow cytometry (concentration of Ce6 = 1 µg/mL).


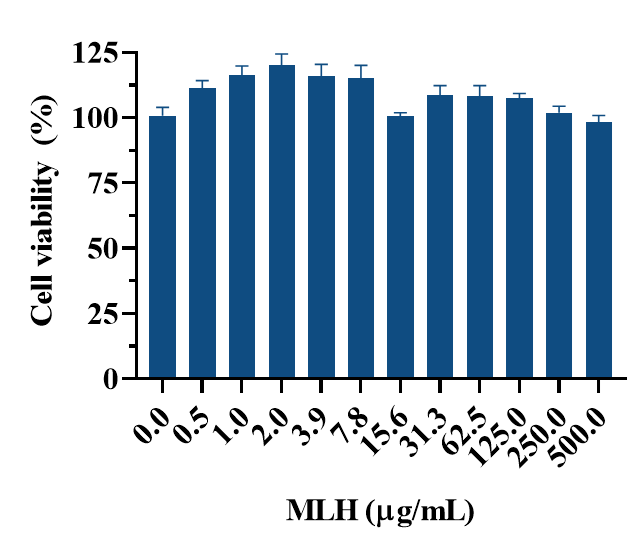


**Fig. S3.** Cell viability test of MLHs against CT-26 cells at various concentration (incubation time = 24 h).
